# Comparing the Use of Transverse Abdominis Plane Block and Splash Block for Postoperative Pain Control in Dogs Undergoing Mastectomy—A Blinded Randomized Prospective Clinical Study

**DOI:** 10.3390/ani15091323

**Published:** 2025-05-02

**Authors:** Daniele Corona, Simone K. Ringer, Stefanie Keller, Iris M. Reichler, Regula Bettschart-Wolfensberger, Annette P. N. Kutter

**Affiliations:** 1 Department of Clinical Diagnostics and Services, Section of Anaesthesiology, Vetsuisse Faculty, University of Zurich, 8057 Zurich, Switzerland; sringer@vetclinics.uzh.ch (S.K.R.); rbettschart@vetclinics.uzh.ch (R.B.-W.); akutter@vetclinics.uzh.ch (A.P.N.K.); 2 Clinic for Animal Reproduction Medicine, Vetsuisse Faculty, University of Zurich, 8057 Zurich, Switzerland; stefi_keller@hotmail.com (S.K.); iris.reichler@uzh.ch (I.M.R.)

**Keywords:** neoplasia, mammary tumor, canine, locoregional anesthesia, pain score

## Abstract

To compare the use of postoperative analgesia for mastectomy, 44 dogs were randomly allocated to either the Splash treatment group (group A) or the Transverse Abdominis Plane block treatment group (TAP, group B). Following intramuscular (IM) premedication with pethidine (4 mg kg^−1^) and acepromazine (0.01 mg kg^−1^), anesthesia was induced with intravenous (IV) propofol and maintained with isoflurane by an anesthetist (DC) who was unaware of the treatment. In group A, ropivacaine 0.5% (2 mg kg^−1^) was administered prior to surgical wound closure. In group B, ropivacaine 0.5% (0.8–1 mg kg^−1^ per point) was administered by ultrasound-guided TAP block with two injection points per treated body side. At the end of the surgery, all dogs received pethidine (4 mg kg^−1^ IM), meloxicam (0.2 mg kg^−1^ IV), and acepromazine (0.005 mg kg^−1^ IV). The animals’ pain was assessed by the anesthetist, who remained unaware of the treatment type used, via the Short Form of the Glasgow Composite Pain Scale. When the pain scores were ≥6, methadone (0.2 mg kg^−1^ IV) and gabapentin (10 mg kg^−1^ per oral) were started. When the pain score remained ≥ 6, ketamine (1 mg kg^−1^ subcutaneously) was administered. The dogs in the TAP block group had lower postoperative pain scores 3–12 h after anesthesia administration was terminated and required significantly less rescue analgesia.

## Simple Summary

Mastectomy for tumour removal is a major surgery which involves a high level of pain in the postoperative phase. As such, postoperative pain reduction with appropriate analgesia is key to animal welfare and positive surgical outcomes. The aim of this study was to evaluate the potential differences in pain control following mastectomy between the Splash block, which consists of topical application of local anaesthetic (LA) directly to surgical site prior to the closure of upper tissue layers, and the Transverse Abdominis Plane (TAP) block, which entails injecting LA into the fascial plane situated between the internal abdominal oblique and transversus abdominis muscles. TAP block exerted in a longer-lasting effect, and was associated with a reduced need for rescue analgesia with opioids and ketamine.

## 1. Introduction

As in case of humans, benign and malignant mammary gland tumors are quite common in cats and dogs [1]. Some European studies showed an incidence of approximately 200 per 100,000 dog-years [2,3,4]. Thethe inguinal pairs are most commonly affected, while the axillary mammary glands are rarely involved at first occurrence. Innervation of the mammary gland in dogs is complex and consists of nerves containing sympathetic and sensory efferent fibres. In female dogs, the mammary gland is innervated by branches of the intercostal and genitofemoral nerves. The cranial thoracic mammary gland receives its nerve supply from the lateral cutaneous branches of the fourth, fifth, and sixth thoracic ventral nerves (intercostal). The caudal thoracic gland is innervated by lateral cutaneous branches of the sixth and seventh thoracic ventral nerves (intercostal). The abdominal and inguinal mammary glands are innervated by the genitofemoral nerve and the ventral cutaneous branches of the first three lumbar nerves (cranial iliohypogastric, caudal iliohypogastric, and ilioinguinal) [5].

Surgery is the treatment of choice for most mammary tumors. The anaesthesia administered during a mastectomy can be managed in various ways. The complexity and the extension of the innervated area make pain management challenging. To provide analgesia, multimodal approaches are used to reduce the required amount of each individual drug and to make use of the beneficial effects of synergistic actions. Local anesthetic agents (LAs) are often used in both animals and humans to provide perioperative analgesia as part of a multimodal approach for pain management. Different locoregional analgesic techniques have been reported in the veterinary medicine literature to address this issue, including epidural injections, the paravertebral (PVT) block, transversus abdominis plane (TAP) block, and Splash block [6,7,8]. Regional anesthesia plays an important role in the positive outcome of human patients [9,10], and inadequate pain management can lead to increased susceptibility to complications, prolonged healing times, impaired mobility, and decline in overall health and quality of life [11,12,13]. In dogs undergoing ovariohysterectomy, better analgesia management in the perioperative period had a positive impact on recovery time and reduced postoperative complications [14]. Postsurgical complications related to inadequate pain management have negative consequences for animal welfare and hospital performance, leading to extended stays and readmissions, both of which are associated with increased costs.

TAP block, combined with intercostal nerve blocks for surgeries such as radical mastectomy, provides intraoperative anti-nociception and short-term postoperative analgesia for dogs undergoing mastectomy [15,16]. To perform such blocks safely, the ultrasound machine must be operated by a knowledgeable person who is familiar with the dog’s local anatomy, the area must be surgically prepared; and it is necessary to set aside sufficient time to implement the block. On the other hand, Splash block is simple to perform and less time-consuming, which may be advantageous in clinical veterinary practice. It consists of topical application of the LA directly to the surgical site prior to the closure of the upper tissue layers [17,18,19,20,21]. We elected to compare those two techniques in a clinical study.

The objective of this study was to compare postoperative pain levels, measured using the Short Form-Glasgow Composite Pain Scale (SF-GCPS) [22] in dogs undergoing mastectomy. This comparison focused on two distinct locoregional anesthetic techniques: the TAP block and the Splash block, both of which were administered with ropivacaine 0.5%. The hypothesis was that the TAP block would enhance intraoperative analgesia, resulting in lower pain scores and a diminished need for rescue analgesia in the postoperative period compared to the Splash block.

## 2. Materials and Methods

This blinded randomized prospective clinical study was approved by the animal experimentation committee of the Canton of Zurich (ZH 145/18). Signed owner’s consent was obtained from the owners of all dogs. A power analysis was performed using an online sample size calculator (https://clincalc.com/stats/samplesize.aspx, accessed on 10 May 2018). A sample size of 44 dogs was selected to demonstrate that the incidence of pain requiring rescue analgesia within 12 h of anesthesia cessation can be reduced from 80% to 40% with a power of 80% and an alpha error of 0.05.

Dogs of all ages, health statuses, and weights undergoing mastectomy (in some instances combined with ovariohysterectomy (OHE)) at the Clinic for Animal Reproduction Medicine of Vetsuisse Faculty Zurich were included in our study study. All patients were randomly assigned to one of two groups, in accordance with randomized table obtained using a randomisation programme (https://www.random.org, accessed on 21 September 2018). Group A received a Splash block and Group B received a TAP block ± intercostal blocks.

Physical examination, complete blood count, serum biochemistry profile, and CT scan were performed in all subjects 2–7 days prior to surgery, and the dogs were allocated to an ASA category. All dogs were physically reevaluated by the anesthetist in charge (anesthetist 1, D.C.) on the morning of the surgery and a underwent a premedication (T0) pain assessment recorded as a a Short Form-Glasgow Composite Pain Score (SF-GCPS). Premedication consisted of intramuscular (IM) administration of pethidine (Pethidine 50 mg mL^−1^; Streuli pharma AG–Uznach, Switzerland) (4 mg kg^−1^) and acepromazine (Prequillan 10 mg mL^−1^; Fatro S.p.A.—Ozzano Emila, Italy) (0.01 mg kg^−1^). After 15 min, an intravenous (IV) catheter was placed in a cephalic vein and after the initial heart rate (HR) measurement (Cardiocap/5,Anadic Medical Systems—Feuerthalen, Switzerland) and non-invasive blood pressure (NIBP) (DINAMAP technology V100—Anadic Medical Systems—Feuerthalen, Switzerland), anesthesia was induced with propofol (Propofol 50 mL (10 mg mL^−1^); Fresenius Kabi, Krieus, Switzerland) IV to effect (total dose 2–5 mg kg^−1^). Endotracheal intubation was performed in the sternal position, and the patient was attached to an anesthetic machine (Aespire View—Anandic Medical Systems—Feuerthalen, Switzerland). Anesthesia was maintained with isoflurane (Isoflo 250 mL; Zoetis—Delemont, Switzerland) in oxygen/air with a total fresh gas flow rate of 10 mL kg^−1^ min^−1^. Anesthesia was administered according to a predetermined anesthesia management chart (Appendix A), always by the same anesthetist (D.C.), who was blinded to the animals’ assigned treatment groups. All dogs were mechanically ventilated to maintain an expired fraction of carbon dioxide (FE’CO_2_) between 35 and 45 mmHg, peak intrapulmonary pressure of 7–15 cmH_2_O, an I:E ratio of 1:2, and zero positive end expiratory pressure. Ringer acetated solution (RA) (Ringer Acetat Bichsel; Laboratorium Dr G. Bichsel AG—Untersee, Switzerland) was administered IV at an initial rate of 5 mL kg^−1^ h^−1^. In the event of hypotension, additional boluses of RA at 5 mL kg^−1^ IV were provided. If the hypotension (MAP < 70 mmHg) persisted, a vasoactive amine- either dobutamine or dopamine—was initiated at a dosage of 0.005 mg kg^−1^ min^−1^ IV, which was adjusted as necessary to maintain a mean arterial blood pressure (MAP) of > 70 mmHg as predetermined by the anesthesia management chart (Appendix A). Body temperature was maintained between 37 and 38.5 °C using warm water mattresses (HIRTZ—Aquatherm 660; Nufer Medical—Bern, Switzerland] and warm air devices (Bair Hugger, Modell 505; Augustine Medical SA—Wakefield, UK]. An arterial catheter was placed in a dorsal pedal artery to monitor invasive mean arterial blood pressure (iMAP). The catheter was connected to a pre-calibrated transducer, which was zeroed to atmospheric pressure and positioned at the level of the right atrium. Heart rate (HR), iMAP, FE’CO_2_, end-tidal isoflurane concentration (FE′ISO), esophageal temperature, and pulse oximetry (SpO_2_) were continuously monitored using a multiparameter monitor and recorded every five minutes (min). Fentanyl (Fentanyl 0.5 mg 10 mL^−1^; SINTETICA S.A.—Mendrisio, Switzerland) was administered during surgery in case of nociceptive response, in accordance with the anesthesia management chart (Appendix A).

Briefly, the nociceptive response was defined as increase in HR and MAP of >20% from the pre-incisional values and/or fighting against the ventilator and/or increased jaw tone/evident palpebral reflex.

The entire abdominal wall and the thoracic wall, in case of a radical mastectomy, were clipped and the skin was aseptically prepared. At this point, the anesthetist (D.C.) was asked to leave the room for 20 min, during which a second anesthetist was responsible for the anesthesia and performed the TAP block ± intercostal blocks in group B. This anesthetist also prepared a syringe with ropivacaine 0.5% for group A or for concurrent sham treatment of group B, a syringe with sodium chloride 0.9% (NaCl 0.9%) (Natrium Chloratum 0.9% Bichsel 100 mL; Grosse Apotheke Dr G. Bichsel AG—Interlaken, Switzerland).

The TAP block was performed with dogs in lateral recumbency, with the side to be blocked facing upward. An ultrasound machine (ACUSON Freestyle—Siemens—Zurich, Switzerland) with a 12 MHz linear probe was used to identify the muscular layers forming the abdominal wall to perform the TAP block as described elsewhere [10]. Briefly, the US transducer was positioned in the dorsal abdominal flank, perpendicularly to the body’s long axis, and cranially to the iliac crest. The transducer was gently slid into position until the three layers of the abdominal wall, the transversus abdominis muscle, the internal oblique abdominal muscle, and the external oblique abdominal muscle were identified. A 22 gauge 40–70 mm spinal needle (Quincke needle 22G × 1^1/2”^ or 22G × 3^1/2”^; P.J. Dahlhause & Co. GmbH Brandneu seit 1854—Köln, Germany) connected to a 15 cm-long tubular extension (Syramed Extension line, Ø 1.5 × 2.5, 15 cm, DEHP-Free PVC, Fingersafe injection Port, Anti-Reflux Valve, Slide Clamp, Female and Male Luer Lock, Arcomed ag, Kloten, Switzerland) pre-filled with the local anesthetic was inserted into a dorso-ventral direction, perpendicular to the body long axis, using an “in-plane” technique. Thus, the needle tip was advanced under ultrasound guidance through the external layers of the abdominal wall toward the fascial plane between the internal abdominal oblique and transversus abdominis muscles (TAP). Ropivacaine 0.5% was used to perform both blocks (TAP or Splash), and NaCl 0.9% was used to dilute it from its original concentration of ropivacaine 0.75% (Ropivacain Sintetica 7.5 mg/mL; Sintetica S.A.—Mendrisio, Switzerland) immediately prior to surgery.

All dogs in the TAP block group received two injections per side, one in the caudal part of the middle abdominal region, cranially to the iliac crest, and a second in the cranial part of the middle abdominal region, caudally to the last rib. In case of unilateral radical mastectomy, the TAP block was performed by injecting 2 × 0.8 mg kg^−1^ of ropivacaine 0.5%, corresponding to 0.16 mL kg^−1^ per point and intercostal nerve blocks (T4–T11) were performed with ropivacaine 0.5% (9 × 0.04 mg kg^−1^ corresponding to 0.08 mL kg^−1^ per point) to complete the block, using a blind intercostal nerve block approach [23]. In case of regional inguinal mastectomy (on the left or right side), a TAP block was performed by injecting 2 × 1 mg kg^−1^ of ropivacaine 0.5%, corresponding to 0.2 mL kg^−1^ per point. In dogs in which mastectomy was associated with ovariohysterectomy, local Splash block with lidocaine 2% (Lidocain HCl Bichsel 20 mg mL^−1^; Grosse Apotheke Dr G. Bichsel AG—Interlaken, Switzerland) (0.5 mg kg^−1^) on the suspensory ligament of the ovary and intraperitoneal ropivacaine 0.5% (2 mg kg^−1^) was used to provide intraoperative analgesia.

In the theatre, when the neoplastic tissue was completely removed, the preprepared syringe containing the LA solution (2 mg kg^−1^) (group A) or the same volume of NaCl 0.9% (group B) was spread on the surgical wound, and surgeons were not permitted to remove it with sponges.

After wound closure, the dogs received pethidine (4 mg kg^−1^) IM and meloxicam (Metacam; Boehringer-Ingelheim—Basel, Switzerland) (0.2 mg kg^−1^) IV. After that, while the animals were still under anesthesia, a compressive bandage, which was not removed until discharge, was applied to reduce the possibility of seroma. As soon as the dogs were extubated, IV acepromazine (0.005 mg kg^−1^) was administered to avoid dysphoria post anesthesia and to ensure a calm recovery. Postoperative pain was evaluated using an SF-GCPS 30 and 60 min after the end of surgery and thereafter every hour up to the 12 h mark by the same anesthetist (anesthetist 1, D.C.) who was blinded to treatment. If SF-GCPS ≥ 6, rescue analgesia with methadone (Methadone 10 mg mL^−1^; Streuli pharma AG, Uznach, Switzerland) (0.2 mg kg^−1^) IV every 4 h and gabapentin (Gabapentin—Mepha 100–50 mg; Mepha—Pharma AG—Basel, Switzerland) (5–10 mg kg^−1^) per oral (PO) every 8 h was started and the animals were excluded from further study with relevant pain scoring. Nevertheless, those dogs were evaluated again, one hour after the administration of rescue analgesia. In case more analgesia was needed, ketamine (Ketanarkon 100; Streuli S.A.—Uznach, Switzerland) (1 mg kg^−1^) was administered subcutaneously (SC) every 6 h. The relevant study observations were stopped in all dogs 12 h after the end of anesthesia. The morning following surgery, patients were reevaluated by the anesthetist. The healing of the surgical wound of each animal was also evaluated for each case by the surgery team, who were blind to the animals’ assigned groups.

### Statistics

Continuous variables (body weight, duration of anesthesia, duration of surgery, and time to first methadone administration from ropivacaine) were analyzed with a *t*-test, while categorical variables such as type of surgery (op_type). fentanyl intra-operation (fenta) and intraperitoneal LA (ipLA) were analyzed with a chi-square test. Intraoperative parameters and drug usage were analyzed with a Local Polynomial Regression Fitting (loess). Pain scores were analyzed based on trajectories and the modelling approach. Differences were considered statistically significant for *p* values < 0.05.

## 3. Results

We observed a good balance between the groups on for all covariates (Table 1). The results are reported as the total number of patients (%) and the mean (SD). Our data were tested for balance checks to establish that the randomization was successful and that there was no significant difference in the distribution of all variables.

### 3.1. Animals, Time Intervals, and Surgical Duration

A total of 44 female dogs were anesthetized and were subsequently allocated into two groups, with 23 dogs in group A and 21 dogs in group B. Two dogs from group A were excluded (one due to intraoperative surgical complications which meant that the study protocol could not be, while the second was, incorrectly, included in the study twice due to a bilateral mastectomy). As such, the final data analysis comprised 42 dogs.

The Splash group included 19/21 dogs classified as ASA 2 and 2/21 dogs classified as ASA 3, whereas, the TAP group comprised 18/21 dogs classified as ASA 2 and 3/21 dogs classified as ASA 3.

The body weights of the dogs enrolled in the study ranged from 3.6 to 37.5 kg [group A 15.7 (8.6), group B 15.2 (9.5)], and there was no significant difference (Table 1).

The anesthesia and surgery times did not differ significantly between the groups (Table 1).

There was no variance in the distribution of surgery type between groups. In total, 12 of the 42 dogs underwent OHE within the same surgical period (Table 1).

The total doses of ropivacaine in group A were as follows, 4 mg kg^−1^ in 9 dogs (with combined surgeries), and 2 mg kg^−1^ in 12 dogs (which underwent unilateral or monoregional mastectomy). Ten of the dogs in group B received dosages of 4 mg kg^−1^, while 11 dogs in this group received 2 mg kg^−1^ doses, respectively.

### 3.2. Monitored Parameters and Drugs Used During Anesthesia

**Figure 1:**
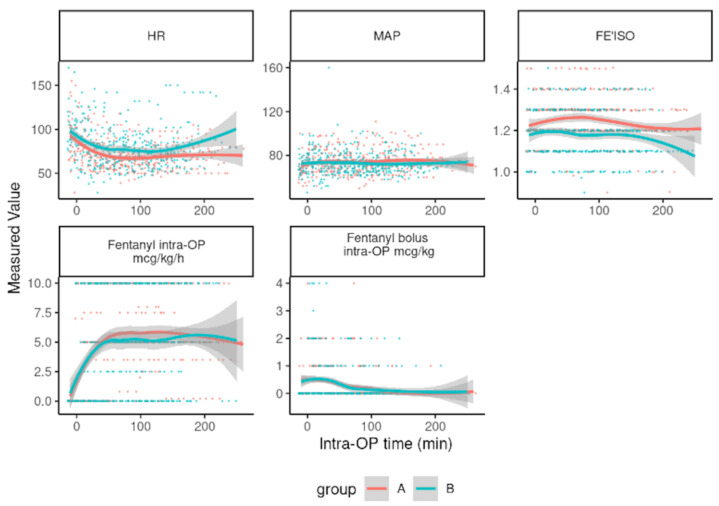
Intraoperative heart rate (HR), invasive mean arterial blood pressure (MAP), expiratory fraction of isoflurane (FE’ISO) in group A-Splash block (21 dogs), and group B-TAP block (21 dogs) from −10 min prior to the start of surgery to the end of surgery. Intraoperative fentanyl infusion dose (mcg kg^−1^ h^−1^) and fentanyl bolus (mcg kg^−1^) in group A-Splash block and group B-TAP block from the beginning of anesthesia to 4 hrs of anesthesia. Time trends show individual data points and Local Polynomial Regression (loess) with 95% CIs shown in the shaded area. If the 95% CI do not overlap, this can be interpreted as a significant difference at that time point.

Overall, the intraoperative HR in group A ranged from 39 to 150 beats min^−1^, whereas in group B, it ranged from 45 to 150 beats min^−1^ with a mean (SD) of 73 (18) and 80 (24), respectively. A statistically significant difference was found over time and between the two groups (*p* value < 0.001), with a higher HR identified in group B (Figure 1).

The intraoperative iMAP (from −10 min to start of surgery to the end of surgery) ranged from 50 to 111 mmHg in group A and 49 to 102 mmHg in group B, with means (SD) of 72.8 (10.7) and 74.2 (10.5), respectively. Statistically, there was no significant difference between the two groups (*p* value = 0.074) (Figure 1).

There was no difference in the propofol requirements between groups in relation to the induction dose [group A 3.7 (1.5), group B 3.8 (1.4) mg kg^−1^] and additional boli given during anaesthesia [group A 0.2 (0.4), group B 0.3 (0.6) mg kg^−1^].

The intraoperative FE′ISO (from −10 min after the start of surgery to the end of surgery) showed a statistically significant difference between the two groups, with group B having a lower FE′ISO than group A (*p* value < 0.001) (Figure 1).

There was no difference in fentanyl dosage (infusion and bolus) during anaesthesia. All of the dogs in group A required fentanyl, while 3/21 dogs (14%) in group B did not.

The RA fluid rate was maintained at 5 mL kg^−1^ h^−1^ in all dogs, and there was no difference between groups regarding the number of RA boluses administered. None of the dogs received Voluven during anesthesia.

Dobutamine or Dopamine dosage during anesthesia indicated a range from 2.5 to 10 mcg kg^−1^ min^−1^ between the two groups. Overall, 33/42 dogs needed the administration of a catecholamine (17/21 in the Splash group and 15/21 in the TAP group—Mean (SD) of 3.3 (2.1) and 3.0 (2.1), respectively. Of the two treatments, Dopamine was administered more frequently (62.5%).

However, although no difference was found for the time to methadone administration since cessation of anesthesia (group A 9 (3.4), group B 8.5 (3.7) h, and a significant difference (*p* = 0.045) was shown if the time taken until the initial rescue analgesia was calculated from the respective administration time of ropivacaine, group A 9 (3.4), and group B 12 (3.8) h.

**Figure 2:**
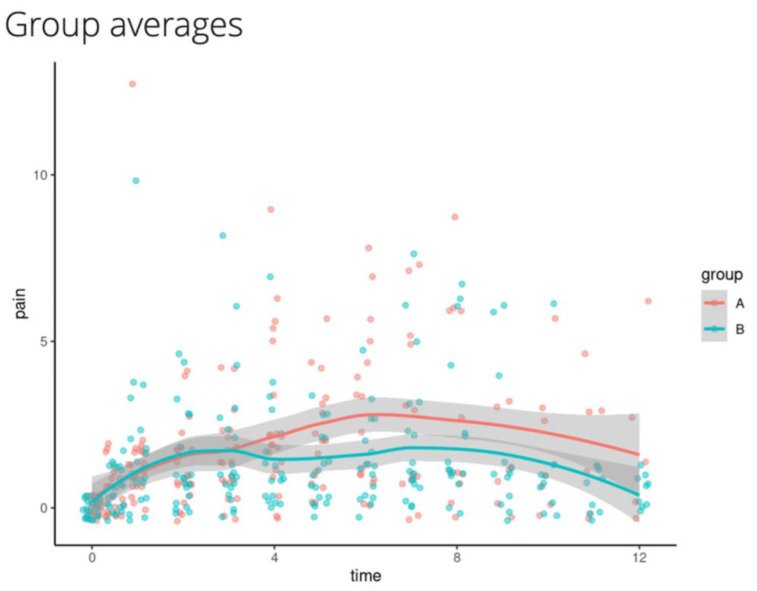
Pain scores in group A-Splash block (21 dogs) and group B-TAP block (21 dogs) over twelve hours post-op. The difference in pain is low for the first 3 h with a parallel development, but then at 3 h+, there is a divergence between the groups. Overall, 95% CI intervals are reported as a shaded area.

Descriptive analysis (Figure 2) 3 h after the cessation of anesthesia indicated a low group difference in pain scores but divergence between the groups with higher pain levels (which nevertheless remained below the rescue threshold) in group A from 3 h on. Group A has had significantly higher pain scores at 6 h postoperative ropivacaine administration.

The number of dogs to be evaluated for pain scoring during the 12 h post-surgery decreased over time as soon as the SF-GCPS was ≥ 6 (Table 2).

Six dogs (29%) in group A were given ketamine (1 mg kg^−1^) SC as additional rescue analgesia when methadone failed to reduce their pain scores within 60 min. In group B, only one dog (5%) was adminitered ketamine as additional rescue analgesia.

## 4. Discussion

The results of our study indicate that both of the described treatment techniques offer effective early postoperative pain management. Despite the relatively low dosage and volume for injection point in the current study compared to previous investigations, dogs treated with TAP blocks experienced less postoperative pain than those receiving Splash block, and the need for rescue analgesia was delayed in the TAP group.

To avoid concurrent drugs affecting the study results, the same protocol was used for all dogs: pethidine, acepromazine and propofol were administered pre- and postoperatively to ensure that the impact on intra- and postoperative pain assessment was minimal. Pethidine is a short-acting (45–60 min) µ-agonist opioid with limited efficacy [24]. Its action during surgical stimulus was presumed to be minimal; when used in combination with acepromazine, it provided reliable sedation for all dogs. Acepromazine is a sedative drug with no analgesic effect, and it was given in tandem with propofol, which was used to induce anesthesia [24]. In the postoperative phase, pethidine was administered at the end of surgery while the animal was still in the surgery room. After that, the dogs were moved to the anesthesia induction area, where a compressive bandage was applied with the animal still under anesthesia. At the initial pain assessment at 30 min after anesthesia cessation, approximately 45 min had elapsed since administration, suggesting that the analgesic effect of pethidine had likely diminished significantly.

In our study, the LA used for the blocks was ropivacaine 0.5%, which reportedly has comparable potency to bupivacaine 0.25% [25]. The total volume of the ropivacaine (0.32 to 0.4 mL kg^−1^) used for the TAP block was lower than the LA volume (bupivacaine) used in a study by Portela et al. (0.87 to 1.06 mL kg^−1^). We chose this lower dose to avoid exceeding the ropivacaine toxic dose (i.e., 4.88 mg kg^−1^) [26] in cases of bilateral mastectomy or association with OHE. However, in Portela et al.’s research, none of the dogs that received more than the maximum recommended dose of 2 mg kg^−1^ of bupivacaine 0.25% [27] exhibited toxic side effects [26,28].

Previous studies showed that implementing a preoperative US-guided TAP block offers the key benefit of intraoperative antinociception [15]. Administering the TAP block prior to surgical insult facilitates pre-emptive analgesia, effectively blocking or modulating pain transmission and helping to prevent or limit central sensitization, which cannot be achieved using the Splash block. While no significant difference was observed in our study in terms of fentanyl administration overall, we noted a tendency toward increased intraoperative fentanyl requirements in dogs which received the Splash block.

In our study, the TAP block appeared to provide complete intra-operative analgesia in only 3/21 dogs, where no fentanyl was necessary to maintain adequate anesthesia. This could potentially be attributed to the relatively low volume used in our experiments. It is possible that in some of the dogs with TAP ± intercostal block, not all nociceptive input was blocked, and therefore the dogs reacted to the surgery and the use of fentanyl was not statistically different between groups. This would eventually confirm that the dosage used in the study was not high enough to result in a complete local block in all dogs tested.

However, it is also important to note that even with an efficient block, fentanyl might still be necessary, particularly when structures in the inguinal canal are ligated (i.e., inguinal vascular structures) or when an additional OHE is performed.

The incomplete intraoperative analgesia effect might also be related to the lower volume used in the present study. This type of fascial block is, by definition, a “volume block”, and a higher volume involving a larger amount of nerve fibres in the fascial plane is required for optimal results. Considering our findings and those reported by Portela et al. [15], which indicated an effective volume of 0.3–0.35 mL/kg per injection point, we theorise that administering this volume of ropivacaine with a lower concentration of 0.25% instead of 0.5% should ensure effective intraoperative and good postoperative analgesia, whilst avoiding exceeding a toxic dose of ropivacaine. Recent studies in the literature have demonstrated that the intraoperative administration of ropivacaine 0.5% exhibits comparable efficacy to that of a lower concentration of 0.3%. However, the postoperative analgesic effect associated with the 0.5% concentration tends to have a longer duration, lasting approximately 11–14 h, in contrast with the more short-lived effect of 8–9 h observed for the 0.3% concentration [29,30].

Notably, the dogs in group B received significantly less isoflurane. Due to the blinded nature of the study, the anesthetist’s provision ofwas based on a predefined algorithm that consisted of increasing the percentage of isoflurane before providing fentanyl if the first intervention was insufficient. Therefore, in the current study, a clear statistical difference between isoflurane is indicative of a more efficient anesthesia regimen, which can only be explained by improved intraoperative analgesia [18,31,32,33,34].

Intraoperative differences in HR were observed, with higher rates recorded in the TAP group. Whether the higher isoflurane dose in group A suppressed the sympathetic tone more efficiently or whether the visual trend toward a higher fentanyl dosage in group Splash (Figure 1) influenced HR cannot be answered within the current study. The individual variations were significant, and at present, discussions regarding whether this was a true group effect or a consequence of individual variations such as breed, age, and type of surgery remain speculative in nature.

Despite the low dose and volume used and relatively long surgery times in our study, the TAP block appeared to provide effective postoperative analgesia, at least within the first few hours post-administration. This finding is consistent with the results of previous studies [8,10,35]. In all of these studies, pain assessment was restricted to within a 2 h period post anesthesia. The groups which received the TAP block achieved lower pain scores with no need for rescue analgesia. In our study, however, we extended the pain assessment period to 12 h post anesthesia. Our findings indicate that the reduction in pain scores persists for several hours (8.5 h) post anesthesia, and thus, the need for rescue analgesia is delayed.

All dogs’ pain levels were assessed by the same anesthetist, who was blind to the treatment provided. There was no significant difference between the two treatments for the first three hours post anesthesia, but a divergence between the groups was evident from the 3-h mark, with higher pain levels (below the rescue threshold) observed in group A from this point on. Group A showed significantly higher pain scores than group B at 6 h postoperative ropivacaine administration.

TAP block plays an interesting role in postoperative analgesia. The long duration of efficient analgesia in 9/21 dogs that did not need rescue analgesia for 12 h is consistent with the existing literature [30,36,37]. This finding significantly enhances our understanding of the superior postoperative analgesic efficacy associated with the block. The TAP block specifically targets the thoracolumbar spinal nerves, which play a crucial role in innervating the abdominal wall muscles. By effectively blocking these nerve pathways, the TAP block provides a profound analgesic effect and extends beyond the immediate surgical site, helping to prevent central sensitization, as previously discussed. In contrast, the Splash block primarily targets the superficial layers of the tissue, specifically the subcutaneous tissue and skin. This limited scope of action may contribute to its reduced effectiveness in managing postoperative pain. By predominantly focusing on these superficial structures, the Splash block may fail to adequately address the deeper nociceptive pathways involved in postoperative pain, resulting in a less comprehensive analgesic effect. Given the extended duration of surgery and anesthesia observed in our study, along with the fact that the TAP block was typically administered 3–4 h prior to the conclusion of the surgical procedure, it is important to consider how these factors may have influenced our findings. The lack of significant differences between the two treatment groups in terms of analgesic outcomes from the cessation of anesthesia to the initial administration of methadone could potentially be attributed to this timing. However, a significant difference was observed for the first postoperative analgesia IV (methadone) if the time was calculated based on the real time of ropivacaine 0.5% administration in both groups.

In our study, another indicator that postoperative analgesia likely improved after a TAP block was our finding that only 1/21 dogs in this group required additional ketamine, while 6/21 dogs in the Splash group received ketamine.

Importantly, we also noted that the performance of the TAP block did not influence the duration of anesthesia between the two groups, underlining that performance of this locoregional anesthesia technique has no major impact on the anesthesia time.

The need for an ultrasound machine and a skilled anesthetist has an undeniable impact on clinical costs, but this burden can be reduced by performing more US-guided blocks over time, improving the quality of intraoperative and postoperative analgesia, and reducing consumption of opioids.

This study had several limitations. The TAP block was performed by different anesthetists, and variances in their skills may have influenced the results. We were unable to judge the block quality after the end of the study as some of the images from the ultrasound blocks were not archived. However, we still identified a difference compared to the Splash block and successfully demonstrated the benefit of the block in the postoperative period. This effect would likely be even greater had the block been implemented exclusively by highly skilled anesthetists.

The surgery was performed by two experienced surgeons with similar technical skills. However, even minimal differences in their techniques may have contributed to variations in the outcome.

An important limitation was that, as per the algorithm, intraoperative fentanyl was only administered after propofol had been provided and the isoflurane concentration increased. Due to the blinded nature of the study, this led to a clear difference in the administration of isoflurane and no significant difference in fentanyl dose, mirroring the fact that the reaction to suboptimal analgesia can be blunted by an increased depth of anesthesia. In future studies, any reaction to surgical stimulation should be immediately treated with antinociceptive drugs.

Another limitation was the inclusion of different types of mastectomy with and without OHE, which might have influenced the results. However, we assigned the same number of OHE patients to each group. The distribution of surgery type also appeared to be balanced (Table 1). We cannot exclude the presence of bias, but the severity of surgical intervention did not appear to be inferior in group B compared to group A.

Additional prospective studies are needed to determine optimal local anaesthetic dosing regimens and concentrations while evaluating their potential impact on reducing intra- and postoperative opioid requirements.

## 5. Conclusions

The current study showed that both TAP ± intercostal block and Splash block with 0.5% ropivacaine provided effective regional analgesia in the postoperative period in dogs undergoing mastectomy. However, the TAP block appeared to provide superior analgesia in the postoperative period, and its duration of action after ropivacaine administration was longer comparable to the Splash block. Nevertheless, Splash is recommended as a good option in the event that no US equipment is available.

## Figures and Tables

**Table 1:** Balance checks: Covariates evaluated in 21 dogs from each group, reported as mean (SD) for weight, duration of surgery (duration_op), anaesthesia duration (duration_ana), and total number of animals (%) for surgery_type (op-type), fentanyl intraop (fenta), and intraperitoneal local anaesthetic administration (ipLA). Legend of op-type: L + RIM (left + right inguinal mastectomy), LIM (left inguinal mastectomy), LRM (left radical mastectomy), RIM (right inguinal mastectomy), RRM (right radical mastectomy), and OHE (ovariohysterectomy).

Label	Levels	A	B	*p*
Total N (%)		21 (50.0)	21 (50.0)	
weight	Mean (SD)	15.7 (8.6)	15.2 (9.5)	0.853
op_type	L + RIM	1 (4.8)	1 (4.8)	0.856
	LIM	0 (0.0)	1 (4.8)	
	LRM	4 (19.0)	4 (19.0)	
	LRM + OHE	4 (19.0)	3 (14.3)	
	LRM + RIM	2 (9.5)	3 (14.3)	
	RIM	1 (4.8)	1 (4.8)	
	RRM	7 (33.3)	5 (23.8)	
	RRM + LIM + OHE	0 (0.0)	2 (9.5)	
	RRM + OHE	2 (9.5)	1 (4.8)	
duration_op	Mean (SD)	170.8 (44.7)	159.8 (50.0)	0.455
duration_ana	Mean (SD)	275.6 (39.4)	273.5 (60.5)	0.903
fenta	no	0 (0.0)	3 (14.3)	0.231
	yes	21 (100.0)	18 (85.7)	
ipLA	no	15 (71.4)	15 (71.4)	1.000
	yes	6 (28.6)	6 (28.6)	

**Table 2:** Number of animals evaluated for pain scoring from time 0 (before surgery) to 12 h after surgery. Numbers decrease with time, as after rescue analgesia ceased, dogs were omitted from the study. Group A-Splash block (21 dogs), group B-TAP block (21 dogs).

Group	0	0.5	1	2	3	4	5	6	7	8	9	10	11	12
A	21	21	21	20	20	20	17	16	13	11	7	7	6	5
B	21	21	21	20	20	18	17	17	17	15	12	10	9	9

## Data Availability

The data are available from the corresponding author upon request. Informed owner consent was obtained from all animals involved in the study.
